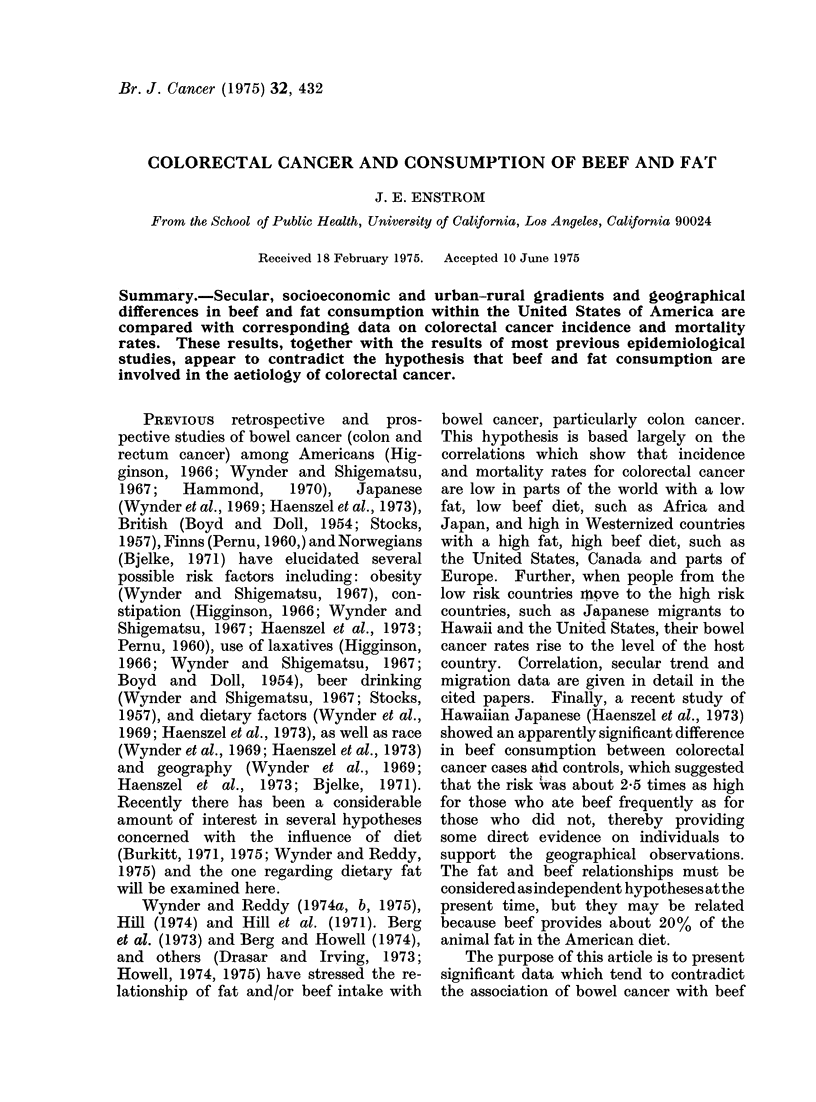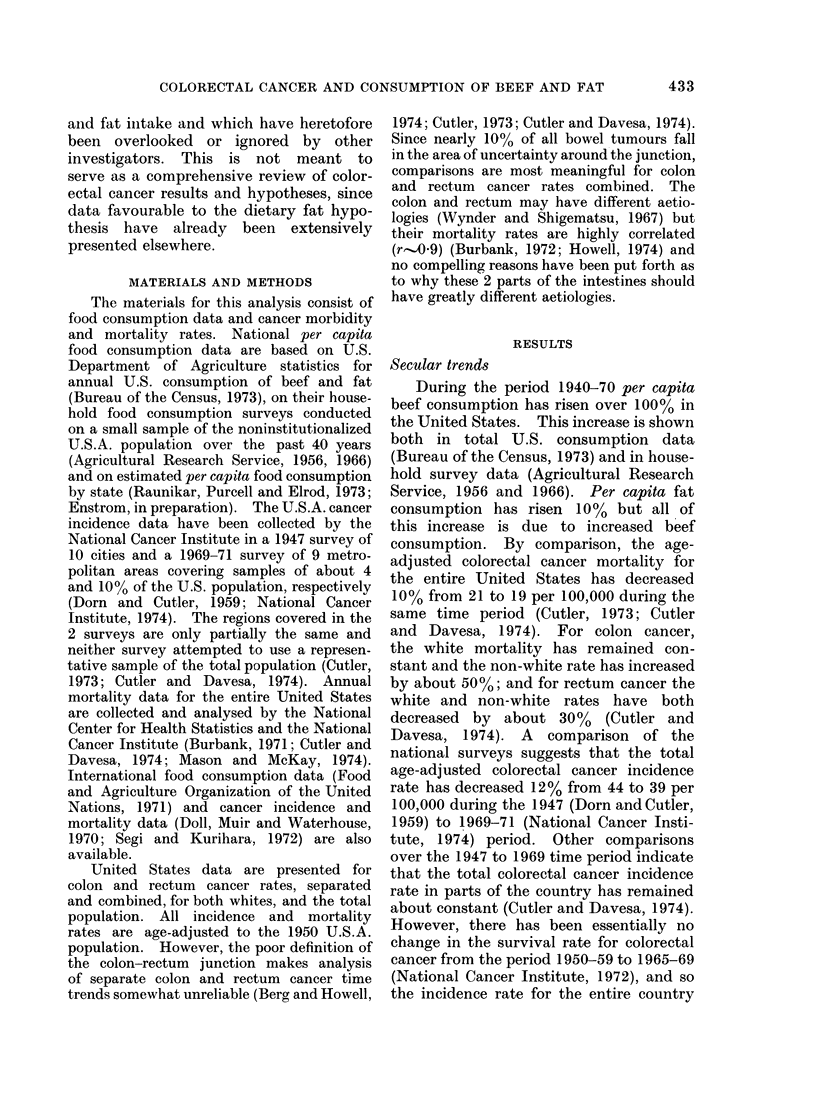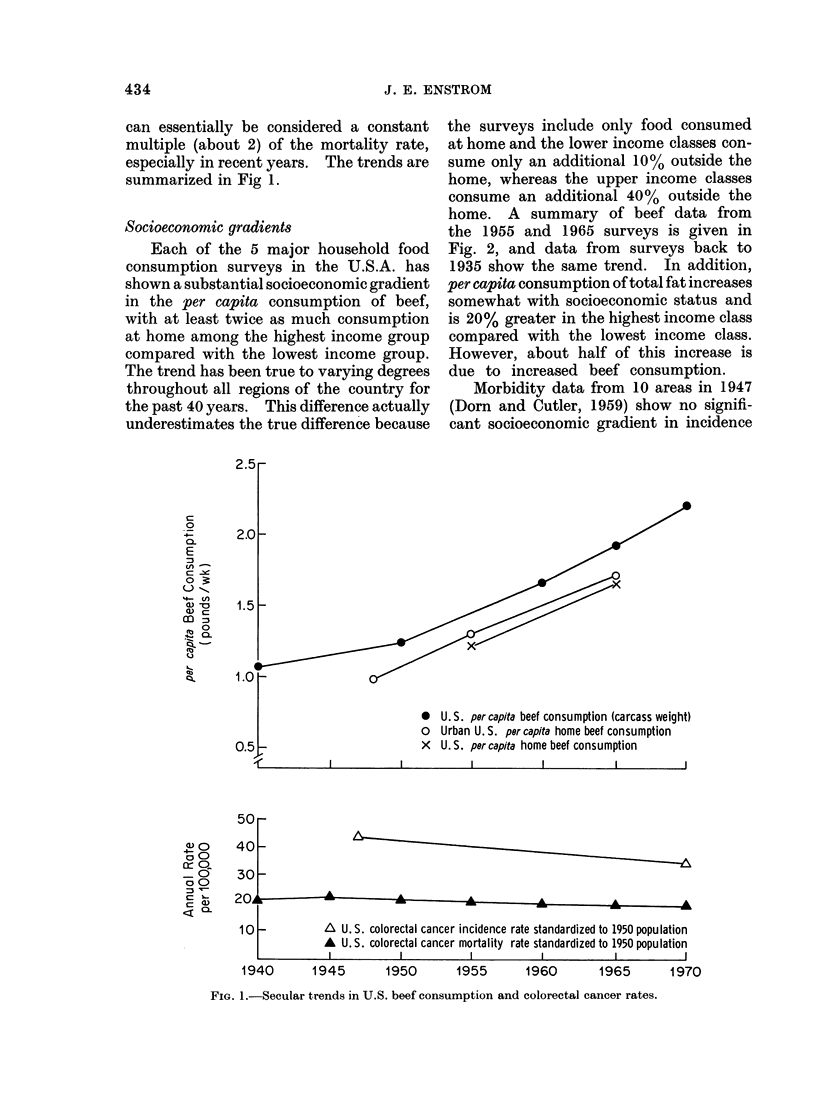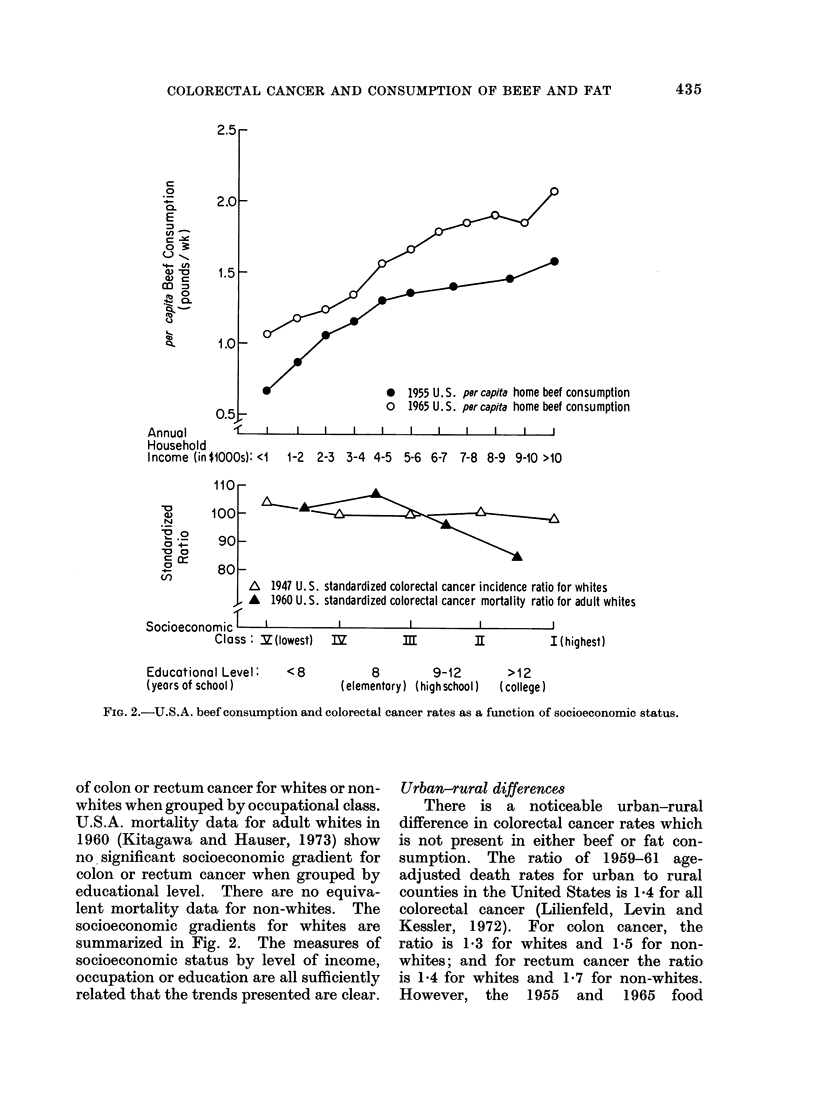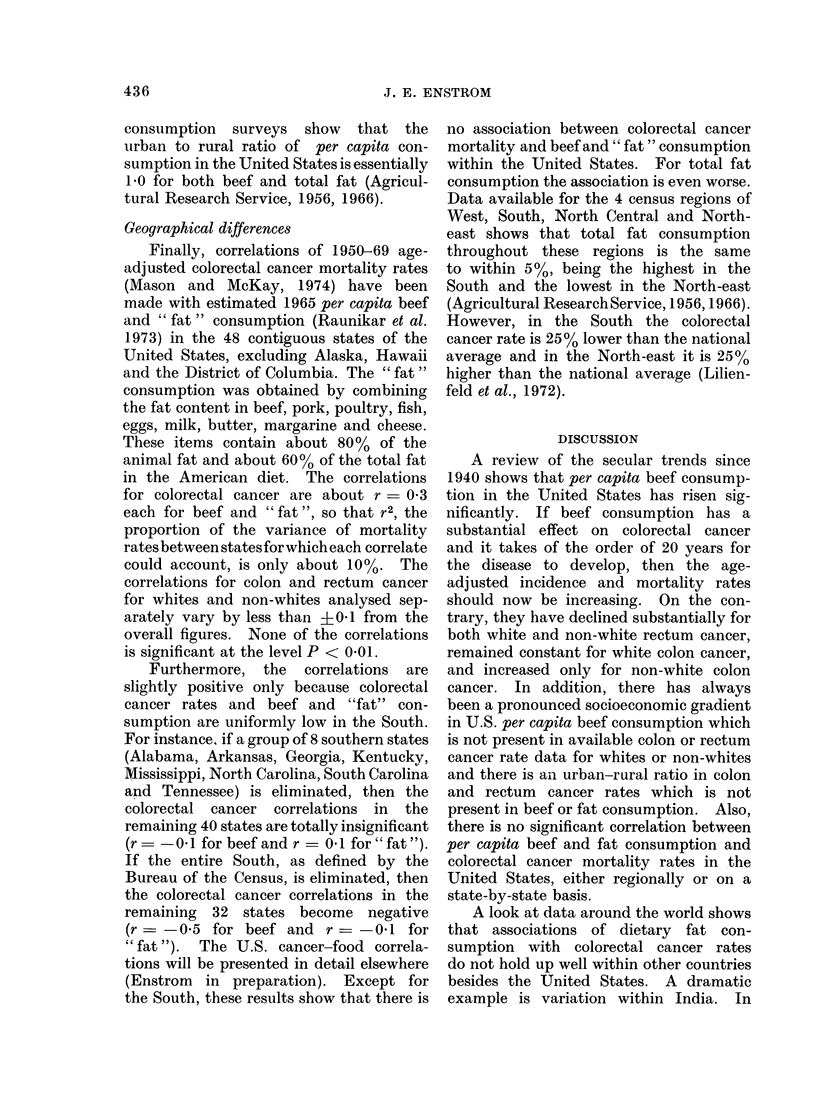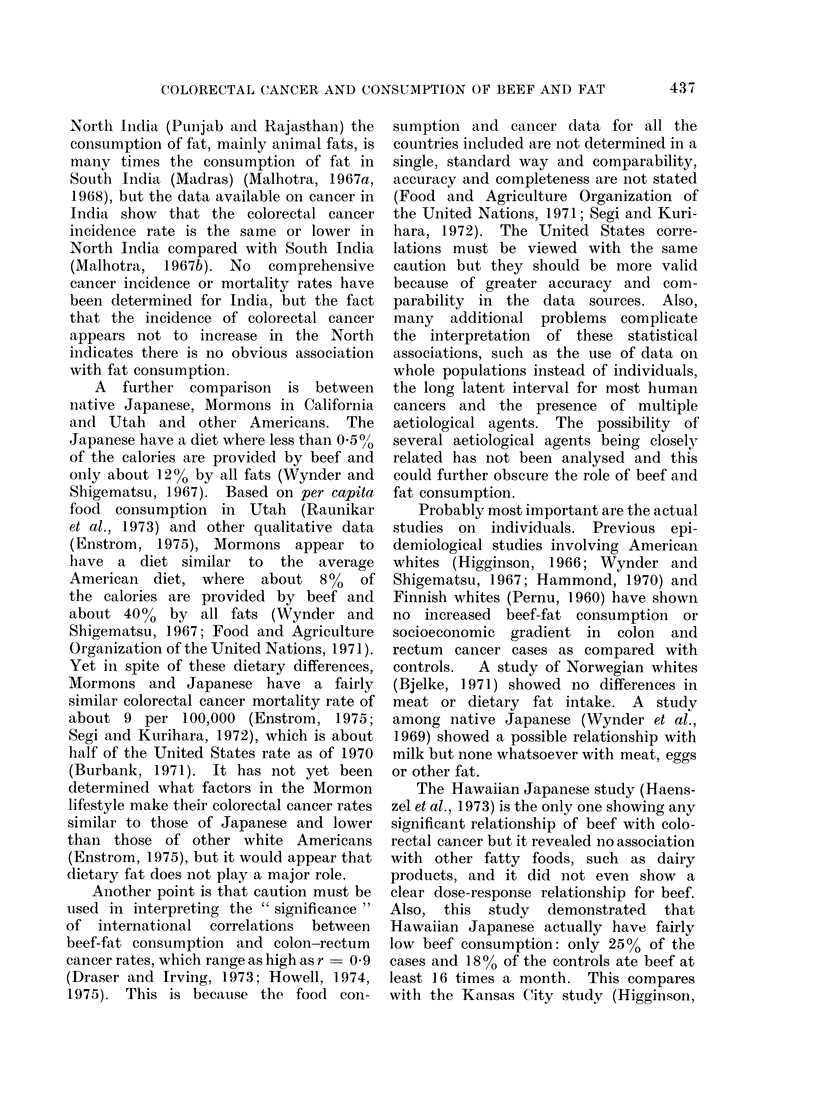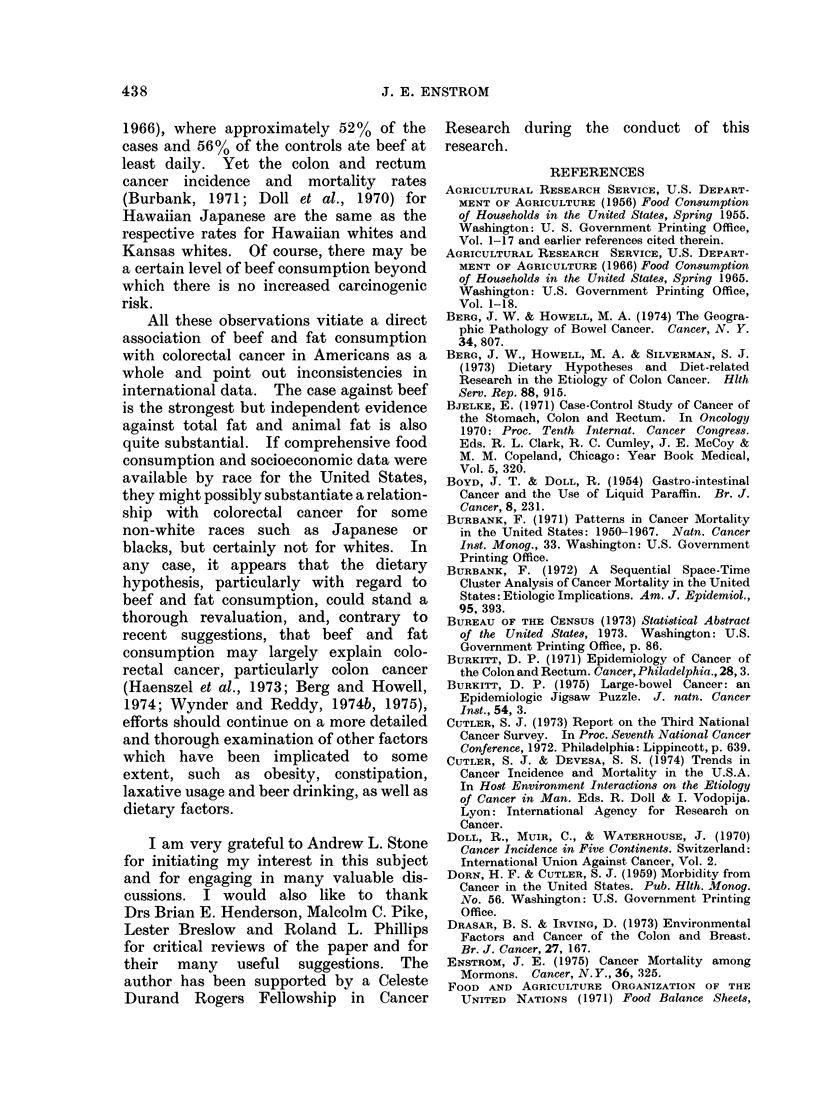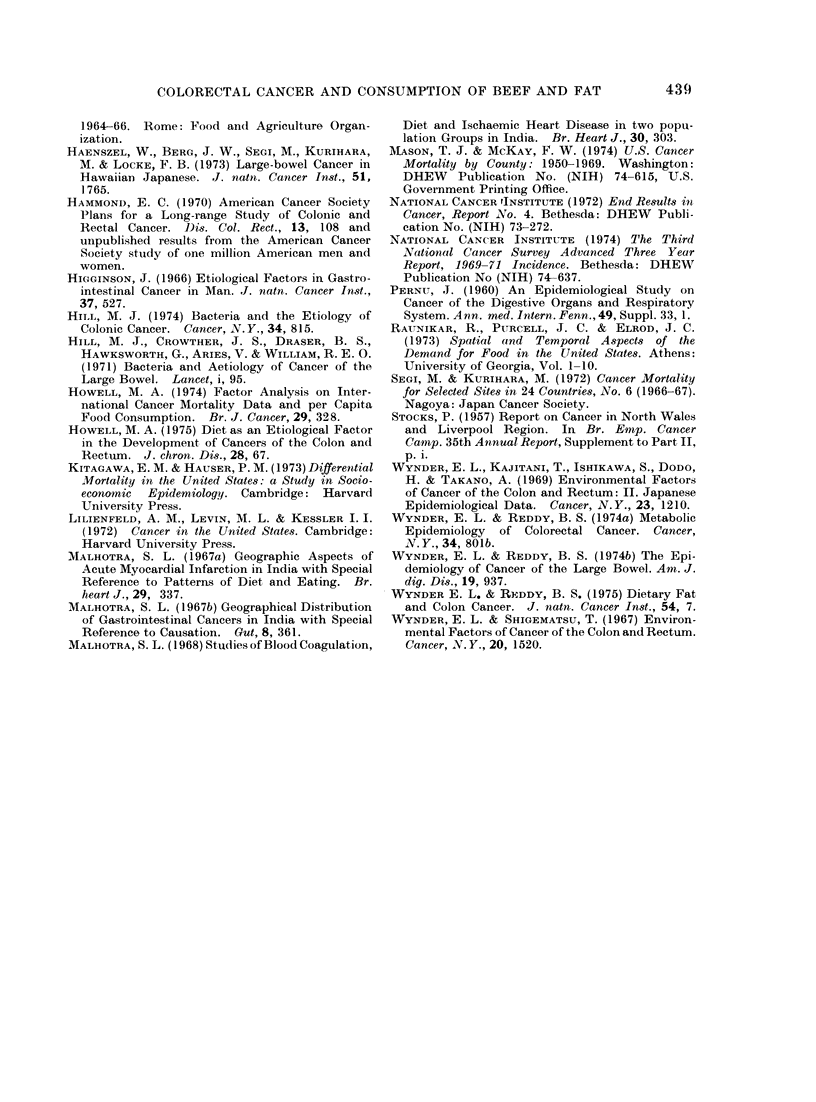# Colorectal cancer and consumption of beef and fat.

**DOI:** 10.1038/bjc.1975.244

**Published:** 1975-10

**Authors:** J. E. Enstrom

## Abstract

Secular, socioeconomic and urban-rural gradients and geographical differences in beef and fat consumption within the United States of America are compared with corresponding data on colorectal cancer incidence and mortality rates. These results, together with the results of most previous epidemiological studies, appear to contradict the hypothesis that beef and fat consumption are involved in the aetiology of colorectal cancer.


*Br. J. Cancer* (1975) **32**, 432

### COLORECTAL CANCER AND CONSUMPTION OF BEEF AND FAT

J. E. ENSTROM

*From the School of Public Health, University of California, Los Angeles, California* 90024

Received 18 February 1975. Accepted 10 June 1975

**Summary.—Secular, socioeconomic and urban–rural gradients and geographical differences in beef and fat consumption within the United States of America are compared with corresponding data on colorectal cancer incidence and mortality rates. These results, together with the results of most previous epidemiological studies, appear to contradict the hypothesis that beef and fat consumption are involved in the aetiology of colorectal cancer.**

PREVIOUS retrospective and prospective studies of bowel cancer (colon and rectum cancer) among Americans (Higginson, 1966; Wynder and Shigematsu, 1967; Hammond, 1970), Japanese (Wynder *et al.*, 1969; Haenszel *et al.*, 1973), British (Boyd and Doll, 1954; Stocks, 1957), Finns (Pernu, 1960,) and Norwegians (Bjelke, 1971) have elucidated several possible risk factors including: obesity (Wynder and Shigematsu, 1967), constipation (Higginson, 1966; Wynder and Shigematsu, 1967; Haenszel *et al.*, 1973; Pernu, 1960), use of laxatives (Higginson, 1966; Wynder and Shigematsu, 1967; Boyd and Doll, 1954), beer drinking (Wynder and Shigematsu, 1967; Stocks, 1957), and dietary factors (Wynder *et al.*, 1969; Haenszel *et al.*, 1973), as well as race (Wynder *et al.*, 1969; Haenszel *et al.*, 1973) and geography (Wynder *et al.*, 1969; Haenszel *et al.*, 1973; Bjelke, 1971). Recently there has been a considerable amount of interest in several hypotheses concerned with the influence of diet (Burkitt, 1971, 1975; Wynder and Reddy, 1975) and the one regarding dietary fat will be examined here.

Wynder and Reddy (1974*a*, *b*, 1975), Hill (1974) and Hill *et al.* (1971). Berg *et al.* (1973) and Berg and Howell (1974), and others (Drasar and Irving, 1973; Howell, 1974, 1975) have stressed the relationship of fat and/or beef intake with bowel cancer, particularly colon cancer. This hypothesis is based largely on the correlations which show that incidence and mortality rates for colorectal cancer are low in parts of the world with a low fat, low beef diet, such as Africa and Japan, and high in Westernized countries with a high fat, high beef diet, such as the United States, Canada and parts of Europe. Further, when people from the low risk countries move to the high risk countries, such as Japanese migrants to Hawaii and the United States, their bowel cancer rates rise to the level of the host country. Correlation, secular trend and migration data are given in detail in the cited papers. Finally, a recent study of Hawaiian Japanese (Haenszel *et al.*, 1973) showed an apparently significant difference in beef consumption between colorectal cancer cases and controls, which suggested that the risk was about 2·5 times as high for those who ate beef frequently as for those who did not, thereby providing some direct evidence on individuals to support the geographical observations. The fat and beef relationships must be considered as independent hypotheses at the present time, but they may be related because beef provides about 20% of the animal fat in the American diet.

The purpose of this article is to present significant data which tend to contradict the association of bowel cancer with beef and fat intake and which have heretofore been overlooked or ignored by other investigators. This is not meant to serve as a comprehensive review of colorectal cancer results and hypotheses, since data favourable to the dietary fat hypothesis have already been extensively presented elsewhere.

### MATERIALS AND METHODS

The materials for this analysis consist of food consumption data and cancer morbidity and mortality rates. National *per capita* food consumption data are based on U.S. Department of Agriculture statistics for annual U.S. consumption of beef and fat (Bureau of the Census, 1973), on their household food consumption surveys conducted on a small sample of the noninstitutionalized U.S.A. population over the past 40 years (Agricultural Research Service, 1956, 1966) and on estimated *per capita* food consumption by state (Raunikar, Purcell and Elrod, 1973; Enstrom, in preparation). The U.S.A. cancer incidence data have been collected by the National Cancer Institute in a 1947 survey of 10 cities and a 1969–71 survey of 9 metropolitan areas covering samples of about 4 and 10% of the U.S. population, respectively (Dorn and Cutler, 1959; National Cancer Institute, 1974). The regions covered in the 2 surveys are only partially the same and neither survey attempted to use a representative sample of the total population (Cutler, 1973; Cutler and Davesa, 1974). Annual mortality data for the entire United States are collected and analysed by the National Center for Health Statistics and the National Cancer Institute (Burbank, 1971; Cutler and Davesa, 1974; Mason and McKay, 1974). International food consumption data (Food and Agriculture Organization of the United Nations, 1971) and cancer incidence and mortality data (Doll, Muir and Waterhouse, 1970; Segi and Kurihara, 1972) are also available.

United States data are presented for colon and rectum cancer rates, separated and combined, for both whites, and the total population. All incidence and mortality rates are age-adjusted to the 1950 U.S.A. population. However, the poor definition of the colon–rectum junction makes analysis of separate colon and rectum cancer time trends somewhat unreliable (Berg and Howell, 1974; Cutler, 1973; Cutler and Davesa, 1974). Since nearly 10% of all bowel tumours fall in the area of uncertainty around the junction, comparisons are most meaningful for colon and rectum cancer rates combined. The colon and rectum may have different aetiologies (Wynder and Shigematsu, 1967) but their mortality rates are highly correlated ($r \sim 0{\cdot}9$) (Burbank, 1972; Howell, 1974) and no compelling reasons have been put forth as to why these 2 parts of the intestines should have greatly different aetiologies.

### RESULTS

### *Secular trends*

During the period 1940–70 *per capita* beef consumption has risen over 100% in the United States. This increase is shown both in total U.S. consumption data (Bureau of the Census, 1973) and in household survey data (Agricultural Research Service, 1956 and 1966). *Per capita* fat consumption has risen 10% but all of this increase is due to increased beef consumption. By comparison, the age-adjusted colorectal cancer mortality for the entire United States has decreased 10% from 21 to 19 per 100,000 during the same time period (Cutler, 1973; Cutler and Davesa, 1974). For colon cancer, the white mortality has remained constant and the non-white rate has increased by about 50%; and for rectum cancer the white and non-white rates have both decreased by about 30% (Cutler and Davesa, 1974). A comparison of the national surveys suggests that the total age-adjusted colorectal cancer incidence rate has decreased 12% from 44 to 39 per 100,000 during the 1947 (Dorn and Cutler, 1959) to 1969–71 (National Cancer Institute, 1974) period. Other comparisons over the 1947 to 1969 time period indicate that the total colorectal cancer incidence rate in parts of the country has remained about constant (Cutler and Davesa, 1974). However, there has been essentially no change in the survival rate for colorectal cancer from the period 1950–59 to 1965–69 (National Cancer Institute, 1972), and so the incidence rate for the entire country can essentially be considered a constant multiple (about 2) of the mortality rate, especially in recent years. The trends are summarized in Fig 1.

*Socioeconomic gradients*

Each of the 5 major household food consumption surveys in the U.S.A. has shown a substantial socioeconomic gradient in the *per capita* consumption of beef, with at least twice as much consumption at home among the highest income group compared with the lowest income group. The trend has been true to varying degrees throughout all regions of the country for the past 40 years. This difference actually underestimates the true difference because the surveys include only food consumed at home and the lower income classes consume only an additional 10% outside the home, whereas the upper income classes consume an additional 40% outside the home. A summary of beef data from the 1955 and 1965 surveys is given in Fig. 2, and data from surveys back to 1935 show the same trend. In addition, *per capita* consumption of total fat increases somewhat with socioeconomic status and is 20% greater in the highest income class compared with the lowest income class. However, about half of this increase is due to increased beef consumption.

Morbidity data from 10 areas in 1947 (Dorn and Cutler, 1959) show no significant socioeconomic gradient in incidence

FIG. 1.—Secular trends in U.S. beef consumption and colorectal cancer rates.

FIG. 2.—U.S.A. beef consumption and colorectal cancer rates as a function of socioeconomic status.

of colon or rectum cancer for whites or non-whites when grouped by occupational class. U.S.A. mortality data for adult whites in 1960 (Kitagawa and Hauser, 1973) show no significant socioeconomic gradient for colon or rectum cancer when grouped by educational level. There are no equivalent mortality data for non-whites. The socioeconomic gradients for whites are summarized in Fig. 2. The measures of socioeconomic status by level of income, occupation or education are all sufficiently related that the trends presented are clear.

*Urban–rural differences*

There is a noticeable urban–rural difference in colorectal cancer rates which is not present in either beef or fat consumption. The ratio of 1959–61 age-adjusted death rates for urban to rural counties in the United States is 1·4 for all colorectal cancer (Lilienfeld, Levin and Kessler, 1972). For colon cancer, the ratio is 1·3 for whites and 1·5 for non-whites; and for rectum cancer the ratio is 1·4 for whites and 1·7 for non-whites. However, the 1955 and 1965 food consumption surveys show that the urban to rural ratio of *per capita* consumption in the United States is essentially 1·0 for both beef and total fat (Agricultural Research Service, 1956, 1966).

*Geographical differences*

Finally, correlations of 1950–69 age-adjusted colorectal cancer mortality rates (Mason and McKay, 1974) have been made with estimated 1965 *per capita* beef and "fat" consumption (Raunikar *et al.* 1973) in the 48 contiguous states of the United States, excluding Alaska, Hawaii and the District of Columbia. The "fat" consumption was obtained by combining the fat content in beef, pork, poultry, fish, eggs, milk, butter, margarine and cheese. These items contain about 80% of the animal fat and about 60% of the total fat in the American diet. The correlations for colorectal cancer are about $r = 0{\cdot}3$ each for beef and "fat", so that $r^2$, the proportion of the variance of mortality rates between states for which each correlate could account, is only about 10%. The correlations for colon and rectum cancer for whites and non-whites analysed separately vary by less than $\pm 0{\cdot}1$ from the overall figures. None of the correlations is significant at the level $P < 0{\cdot}01$.

Furthermore, the correlations are slightly positive only because colorectal cancer rates and beef and "fat" consumption are uniformly low in the South. For instance, if a group of 8 southern states (Alabama, Arkansas, Georgia, Kentucky, Mississippi, North Carolina, South Carolina and Tennessee) is eliminated, then the colorectal cancer correlations in the remaining 40 states are totally insignificant ($r = -0{\cdot}1$ for beef and $r = 0{\cdot}1$ for "fat"). If the entire South, as defined by the Bureau of the Census, is eliminated, then the colorectal cancer correlations in the remaining 32 states become negative ($r = -0{\cdot}5$ for beef and $r = -0{\cdot}1$ for "fat"). The U.S. cancer–food correlations will be presented in detail elsewhere (Enstrom in preparation). Except for the South, these results show that there is no association between colorectal cancer mortality and beef and "fat" consumption within the United States. For total fat consumption the association is even worse. Data available for the 4 census regions of West, South, North Central and North-east shows that total fat consumption throughout these regions is the same to within 5%, being the highest in the South and the lowest in the North-east (Agricultural Research Service, 1956, 1966). However, in the South the colorectal cancer rate is 25% lower than the national average and in the North-east it is 25% higher than the national average (Lilienfeld *et al.*, 1972).

### DISCUSSION

A review of the secular trends since 1940 shows that *per capita* beef consumption in the United States has risen significantly. If beef consumption has a substantial effect on colorectal cancer and it takes of the order of 20 years for the disease to develop, then the age-adjusted incidence and mortality rates should now be increasing. On the contrary, they have declined substantially for both white and non-white rectum cancer, remained constant for white colon cancer, and increased only for non-white colon cancer. In addition, there has always been a pronounced socioeconomic gradient in U.S. *per capita* beef consumption which is not present in available colon or rectum cancer rate data for whites or non-whites and there is an urban–rural ratio in colon and rectum cancer rates which is not present in beef or fat consumption. Also, there is no significant correlation between *per capita* beef and fat consumption and colorectal cancer mortality rates in the United States, either regionally or on a state-by-state basis.

A look at data around the world shows that associations of dietary fat consumption with colorectal cancer rates do not hold up well within other countries besides the United States. A dramatic example is variation within India. In

North India (Punjab and Rajasthan) the consumption of fat, mainly animal fats, is many times the consumption of fat in South India (Madras) (Malhotra, 1967*a*, 1968), but the data available on cancer in India show that the colorectal cancer incidence rate is the same or lower in North India compared with South India (Malhotra, 1967*b*). No comprehensive cancer incidence or mortality rates have been determined for India, but the fact that the incidence of colorectal cancer appears not to increase in the North indicates there is no obvious association with fat consumption.

A further comparison is between native Japanese, Mormons in California and Utah and other Americans. The Japanese have a diet where less than 0·5% of the calories are provided by beef and only about 12% by all fats (Wynder and Shigematsu, 1967). Based on *per capita* food consumption in Utah (Raunikar *et al.*, 1973) and other qualitative data (Enstrom, 1975), Mormons appear to have a diet similar to the average American diet, where about 8% of the calories are provided by beef and about 40% by all fats (Wynder and Shigematsu, 1967; Food and Agriculture Organization of the United Nations, 1971). Yet in spite of these dietary differences, Mormons and Japanese have a fairly similar colorectal cancer mortality rate of about 9 per 100,000 (Enstrom, 1975; Segi and Kurihara, 1972), which is about half of the United States rate as of 1970 (Burbank, 1971). It has not yet been determined what factors in the Mormon lifestyle make their colorectal cancer rates similar to those of Japanese and lower than those of other white Americans (Enstrom, 1975), but it would appear that dietary fat does not play a major role.

Another point is that caution must be used in interpreting the " significance " of international correlations between beef-fat consumption and colon–rectum cancer rates, which range as high as $r = 0{\cdot}9$ (Draser and Irving, 1973; Howell, 1974, 1975). This is because the food consumption and cancer data for all the countries included are not determined in a single, standard way and comparability, accuracy and completeness are not stated (Food and Agriculture Organization of the United Nations, 1971; Segi and Kurihara, 1972). The United States correlations must be viewed with the same caution but they should be more valid because of greater accuracy and comparability in the data sources. Also, many additional problems complicate the interpretation of these statistical associations, such as the use of data on whole populations instead of individuals, the long latent interval for most human cancers and the presence of multiple aetiological agents. The possibility of several aetiological agents being closely related has not been analysed and this could further obscure the role of beef and fat consumption.

Probably most important are the actual studies on individuals. Previous epidemiological studies involving American whites (Higginson, 1966; Wynder and Shigematsu, 1967; Hammond, 1970) and Finnish whites (Pernu, 1960) have shown no increased beef-fat consumption or socioeconomic gradient in colon and rectum cancer cases as compared with controls. A study of Norwegian whites (Bjelke, 1971) showed no differences in meat or dietary fat intake. A study among native Japanese (Wynder *et al.*, 1969) showed a possible relationship with milk but none whatsoever with meat, eggs or other fat.

The Hawaiian Japanese study (Haenszel *et al.*, 1973) is the only one showing any significant relationship of beef with colorectal cancer but it revealed no association with other fatty foods, such as dairy products, and it did not even show a clear dose-response relationship for beef. Also, this study demonstrated that Hawaiian Japanese actually have fairly low beef consumption: only 25% of the cases and 18% of the controls ate beef at least 16 times a month. This compares with the Kansas City study (Higginson,

1966), where approximately 52% of the cases and 56% of the controls ate beef at least daily. Yet the colon and rectum cancer incidence and mortality rates (Burbank, 1971; Doll *et al.*, 1970) for Hawaiian Japanese are the same as the respective rates for Hawaiian whites and Kansas whites. Of course, there may be a certain level of beef consumption beyond which there is no increased carcinogenic risk.

All these observations vitiate a direct association of beef and fat consumption with colorectal cancer in Americans as a whole and point out inconsistencies in international data. The case against beef is the strongest but independent evidence against total fat and animal fat is also quite substantial. If comprehensive food consumption and socioeconomic data were available by race for the United States, they might possibly substantiate a relationship with colorectal cancer for some non-white races such as Japanese or blacks, but certainly not for whites. In any case, it appears that the dietary hypothesis, particularly with regard to beef and fat consumption, could stand a thorough revaluation, and, contrary to recent suggestions, that beef and fat consumption may largely explain colorectal cancer, particularly colon cancer (Haenszel *et al.*, 1973; Berg and Howell, 1974; Wynder and Reddy, 1974*b*, 1975), efforts should continue on a more detailed and thorough examination of other factors which have been implicated to some extent, such as obesity, constipation, laxative usage and beer drinking, as well as dietary factors.

I am very grateful to Andrew L. Stone for initiating my interest in this subject and for engaging in many valuable discussions. I would also like to thank Drs Brian E. Henderson, Malcolm C. Pike, Lester Breslow and Roland L. Phillips for critical reviews of the paper and for their many useful suggestions. The author has been supported by a Celeste Durand Rogers Fellowship in Cancer Research during the conduct of this research.

### REFERENCES

Agricultural Research Service, U.S. Department of Agriculture (1956) *Food Consumption of Households in the United States, Spring* 1955. Washington: U. S. Government Printing Office, Vol. 1–17 and earlier references cited therein.

Agricultural Research Service, U.S. Department of Agriculture (1966) *Food Consumption of Households in the United States, Spring* 1965. Washington: U.S. Government Printing Office, Vol. 1–18.

Berg, J. W. & Howell, M. A. (1974) The Geographic Pathology of Bowel Cancer. *Cancer, N. Y.* **34**, 807.

Berg, J. W., Howell, M. A. & Silverman, S. J. (1973) Dietary Hypotheses and Diet-related Research in the Etiology of Colon Cancer. *Hlth Serv. Rep.* **88**, 915.

Bjelke, E. (1971) Case-Control Study of Cancer of the Stomach, Colon and Rectum. In *Oncology* 1970: *Proc. Tenth Internat. Cancer Congress.* Eds. R. L. Clark, R. C. Cumley, J. E. McCoy & M. M. Copeland, Chicago: Year Book Medical, Vol. 5, 320.

Boyd, J. T. & Doll, R. (1954) Gastro-intestinal Cancer and the Use of Liquid Paraffin. *Br. J. Cancer*, **8**, 231.

Burbank, F. (1971) Patterns in Cancer Mortality in the United States: 1950–1967. *Natn. Cancer Inst. Monog.*, 33. Washington: U.S. Government Printing Office.

Burbank, F. (1972) A Sequential Space-Time Cluster Analysis of Cancer Mortality in the United States: Etiologic Implications. *Am. J. Epidemiol.*, **95**, 393.

Bureau of the Census (1973) *Statistical Abstract of the United States*, 1973. Washington: U.S. Government Printing Office, p. 86.

Burkitt, D. P. (1971) Epidemiology of Cancer of the Colon and Rectum. *Cancer, Philadelphia.*, **28**, 3.

Burkitt, D. P. (1975) Large-bowel Cancer: an Epidemiologic Jigsaw Puzzle. *J. natn. Cancer Inst.*, **54**, 3.

Cutler, S. J. (1973) Report on the Third National Cancer Survey. In *Proc. Seventh National Cancer Conference*, 1972. Philadelphia: Lippincott, p. 639.

Cutler, S. J. & Devesa, S. S. (1974) Trends in Cancer Incidence and Mortality in the U.S.A. In *Host Environment Interactions on the Etiology of Cancer in Man.* Eds. R. Doll & I. Vodopija. Lyon: International Agency for Research on Cancer.

Doll, R., Muir, C., & Waterhouse, J. (1970) *Cancer Incidence in Five Continents.* Switzerland: International Union Against Cancer, Vol. 2.

Dorn, H. F. & Cutler, S. J. (1959) Morbidity from Cancer in the United States. *Pub. Hlth. Monog. No.* 56. Washington: U.S. Government Printing Office.

Drasar, B. S. & Irving, D. (1973) Environmental Factors and Cancer of the Colon and Breast. *Br. J. Cancer*, **27**, 167.

Enstrom, J. E. (1975) Cancer Mortality among Mormons. *Cancer, N.Y.*, **36**, 325.

Food and Agriculture Organization of the United Nations (1971) *Food Balance Sheets*,

1964–66. Rome: Food and Agriculture Organization.

HAENSZEL, W., BERG, J. W., SEGI, M., KURIHARA, M. & LOCKE, F. B. (1973) Large-bowel Cancer in Hawaiian Japanese. *J. natn. Cancer Inst.*, **51**, 1765.

HAMMOND, E. C. (1970) American Cancer Society Plans for a Long-range Study of Colonic and Rectal Cancer. *Dis. Col. Rect.*, **13**, 108 and unpublished results from the American Cancer Society study of one million American men and women.

HIGGINSON, J. (1966) Etiological Factors in Gastrointestinal Cancer in Man. *J. natn. Cancer Inst.*, **37**, 527.

HILL, M. J. (1974) Bacteria and the Etiology of Colonic Cancer. *Cancer, N.Y.*, **34**, 815.

HILL, M. J., CROWTHER, J. S., DRASER, B. S., HAWKSWORTH, G., ARIES, V. & WILLIAM, R. E. O. (1971) Bacteria and Aetiology of Cancer of the Large Bowel. *Lancet*, i, 95.

HOWELL, M. A. (1974) Factor Analysis on International Cancer Mortality Data and per Capita Food Consumption. *Br. J. Cancer*, **29**, 328.

HOWELL, M. A. (1975) Diet as an Etiological Factor in the Development of Cancers of the Colon and Rectum. *J. chron. Dis.*, **28**, 67.

KITAGAWA, E. M. & HAUSER, P. M. (1973) *Differential Mortality in the United States: a Study in Socioeconomic Epidemiology*. Cambridge: Harvard University Press.

LILIENFELD, A. M., LEVIN, M. L. & KESSLER I. I. (1972) *Cancer in the United States*. Cambridge: Harvard University Press.

MALHOTRA, S. L. (1967*a*) Geographic Aspects of Acute Myocardial Infarction in India with Special Reference to Patterns of Diet and Eating. *Br. heart J.*, **29**, 337.

MALHOTRA, S. L. (1967*b*) Geographical Distribution of Gastrointestinal Cancers in India with Special Reference to Causation. *Gut*, **8**, 361.

MALHOTRA, S. L. (1968) Studies of Blood Coagulation, Diet and Ischaemic Heart Disease in two population Groups in India. *Br. Heart J.*, **30**, 303.

MASON, T. J. & MCKAY, F. W. (1974) *U.S. Cancer Mortality by County:* 1950–1969. Washington: DHEW Publication No. (NIH) 74-615, U.S. Government Printing Office.

NATIONAL CANCER INSTITUTE (1972) *End Results in Cancer, Report No.* 4. Bethesda: DHEW Publication No. (NIH) 73–272.

NATIONAL CANCER INSTITUTE (1974) *The Third National Cancer Survey Advanced Three Year Report, 1969–71 Incidence.* Bethesda: DHEW Publication No (NIH) 74–637.

PERNU, J. (1960) An Epidemiological Study on Cancer of the Digestive Organs and Respiratory System. *Ann. med. Intern. Fenn.*, **49**, Suppl. 33, 1.

RAUNIKAR, R., PURCELL, J. C. & ELROD, J. C. (1973) *Spatial and Temporal Aspects of the Demand for Food in the United States.* Athens: University of Georgia, Vol. 1–10.

SEGI, M. & KURIHARA, M. (1972) *Cancer Mortality for Selected Sites in* 24 *Countries, No.* 6 (1966–67). Nagoya: Japan Cancer Society.

STOCKS, P. (1957) Report on Cancer in North Wales and Liverpool Region. In *Br. Emp. Cancer Camp.* 35th *Annual Report*, Supplement to Part II, p. i.

WYNDER, E. L., KAJITANI, T., ISHIKAWA, S., DODO, H. & TAKANO, A. (1969) Environmental Factors of Cancer of the Colon and Rectum: II. Japanese Epidemiological Data. *Cancer, N.Y.*, **23**, 1210.

WYNDER, E. L. & REDDY, B. S. (1974*a*) Metabolic Epidemiology of Colorectal Cancer. *Cancer, N.Y.*, **34**, 801*b*.

WYNDER, E. L. & REDDY, B. S. (1974*b*) The Epidemiology of Cancer of the Large Bowel. *Am. J. dig. Dis.*, **19**, 937.

WYNDER E. L. & REDDY, B. S. (1975) Dietary Fat and Colon Cancer. *J. natn. Cancer Inst.*, **54**, 7.

WYNDER, E. L. & SHIGEMATSU, T. (1967) Environmental Factors of Cancer of the Colon and Rectum. *Cancer, N.Y.*, **20**, 1520.